# Cocoa Coproducts-Based and Walnut Oil Gelled Emulsion as Animal Fat Replacer and Healthy Bioactive Source in Beef Burgers

**DOI:** 10.3390/foods10112706

**Published:** 2021-11-05

**Authors:** Carmen Botella-Martinez, Raquel Lucas-González, José M. Lorenzo, Eva María Santos, Marcelo Rosmini, Néstor Sepúlveda, Alfredo Teixeira, Estrella Sayas-Barberá, Jose A. Pérez-Alvarez, Juana Fernandez-Lopez, Manuel Viuda-Martos

**Affiliations:** 1 IPOA Research Group, Agro-Food Technology Department, Centro de Investigación e Innovación Agroalimentaria y Agroambiental (CIAGRO-UMH), Miguel Hernández University, Orihuela, 03312 Alicante, Spain; Carmen.botella@umh.es (C.B.-M.); raquel.lucasg@umh.es (R.L.-G.); estrella.sayas@umh.es (E.S.-B.); ja.perez@umh.es (J.A.P.-A.); j.fernandez@umh.es (J.F.-L.); 2 Centro Tecnológico de la Carne de Galicia, Avd. Galicia No. 4, Parque Tecnológico de Galicia, San Cibrao das Viñas, 32900 Ourense, Spain; jmlorenzo@ceteca.es; 3 Área de Tecnología de los Alimentos, Facultad de Ciencias de Ourense, Universidad de Vigo, 32004 Ourense, Spain; 4 Area Académica de Química, Universidad Autónoma del Estado de Hidalgo, Carr. Pachuca-Tulancingo Km. 4.5, Mineral de la Reforma 42184, Hidalgo, Mexico; emsantos@uaeh.edu.mx; 5 Department of Public Health, Faculty of Veterinary Science, National University of the Litoral, Esperanza 3080, Santa Fe, Argentina; mrosmini@unl.edu.ar; 6 Departamento de Producción Agropecuaria, Facultad de Ciencias Agropecuarias y Forestales, Universidad de La Frontera, Campus Integrado Andrés Bello Montevideo s/n, Temuco 4813067, Chile; nestor.sepulveda@ufrontera.cl; 7 Mountain Research Centre (CIMO), Escola Superior Agrária, Instituto Politécnico de Bragança, Campus Sta Apolónia Apt, 5300-253 Bragança, Portugal; teixeira@ipb.pt

**Keywords:** reformulation, meat products, gelled emulsion, cocoa bean shell, walnut oil, lipidic profile, health indices

## Abstract

The aim of this work was to evaluate the effects on the chemical, physic-chemical, technological, and sensory properties of beef burger when replacing different quantities of fat (50 and 100%) with different levels of oil-in-water-gelled emulsion elaborated with walnut oil and cocoa bean shell flour (GECW). The chemical composition of the samples was affected by the fat replacement. The reformulation increased the moisture and ash content while the fat and protein content decreased with respect to the control sample. The linolenic and linolenic acid content of the beef burgers increased as the GECW replacement was augmented. The polyunsaturated fatty/saturated fatty acid ratio increased in both raw and cooked burgers, whereas the atherogenicity index and thrombogenicity index were reduced in both raw and cooked burgers with respect to the control sample. The use of GECW as a fat replacer was found to be effective in improving the cooking loss. Similarly, there were positive effects on reductions in the diameter and the increases in the thickness of the beef burgers. Regarding lipid stability, in both the raw and cooked burgers, the reformulation increased the 2-thiobarbituric acid reactive substance (TBARs) values with respect to the control sample. In both types of reformulated burgers, three bound polyphenols (mainly catechin and epicatechin) and two free polyphenols were identified, as were methylxanthines theobromine and caffeine. The sensory properties for the control and partial pork backfat replacement treatments were similar, while the sample with the total pork backfat replacement treatment showed the lowest scores. The blend of cocoa bean shell flour and walnut oil could be used as new ingredients for the development of beef burgers with a healthier nutritional profile without demeriting their sensory or cooking characteristics and physic-chemical properties.

## 1. Introduction

Meat and meat products play an essential role in a healthy and well-balanced diet because of their nutritional properties and their great biological importance as an excellent source of proteins; essential amino acids; minerals, including iron, phosphorous, and zinc; as well as vitamins such as niacin, pyridoxine, and cobalamin [1]. Nevertheless, in recent years, many consumers have questioned their high meat intake and have questioned whether the nutritious benefits imparted by meat and meat products are annulled due to these types of products having an excessive animal fat content. Thus, in cooked meat products, the fat content is around 20–35 g/100 g of the product; however, this fat has a high content of saturated fatty acids and is poor terms of its polyunsaturated fatty acid content [2]. The high consumption of these types of products has been associated with the development of several non-communicable chronic diseases, including coronary heart disease, type 2 diabetes, strokes, overweight, and obesity, among the others [3].

The meat industry is sensitive to this problem and is researching and developing different strategies to reduce or replace these potentially harmful fats with other fats with a healthier lipid profile, such as vegetable (chia oil, walnut oil, sesame oil, olive oil, flaxseed oil, etc.) or marine (salmon, algae, etc.) oils [4]. However, it must be acknowledged that fat substitution is a very difficult task since animal fat plays a very important technological role in the flavour, juiciness, textural properties, and cook yield of products [5]. In this way, several strategies to reduce the fat content in meat products are based on (*i*) the direct addition of oils with a healthy lipid profile; (*ii*) the addition of encapsulated oils in different matrices; and (*iii*) the use of structured oils, which include oleogels or gelled emulsions [6].

Among all of these strategies, the use of gelled emulsions has great potential for their use as a fat substitute in meat products. Therefore, several scientific studies have demonstrated the high potential of this type of structured oils as fat replacers [7,8,9,10]. To elaborate, several components of a gelled emulsion could be used; however, oils that show emulsifying and gelling properties are the ones that could be adapted the best for the development of these matrices. In this sense, plant-based products, including vegetable proteins (soy protein, pea protein, etc.), dietary fibres (inulin, pectin, carrageenan, mucilage, etc.), and cereal or pseudocereal flours, could be considered to be a good source of ingredients for the development of these gelled emulsions [11,12,13]. Other possible ingredients for the development of these gelled emulsions are agro-industrial co-products. These co-products have shown very good techno-functional properties, such as emulsifying or gelling properties as well as bioactive compounds, including carotenoids, phenolic acids, and flavonoids, which can improve the lipid stability of the gelled emulsion as well as that of the final food products.

A very interesting co-product is cocoa bean shell because of its high content of bioactive compounds as well as its techno-functional properties. It is possible to find several polyphenolic compounds in its composition, mainly catechin and epicatechin [14], and a high content of methylxanthines, such as theobromine and caffeine [15]. Due to the content of these bioactive compounds as well as to its techno-functional properties, this co-product is a good candidate to act as an emulsifier agent in the development of gelled emulsion. Consequently, the aim of this work was to evaluate the effects on the chemical, physic-chemical, technological, and sensory properties of beef burger when replacing different quantities of fat (50 and 100%) with different levels of gelled emulsion elaborated with walnut oil and cocoa bean shell flour (coproduct for cocoa processing industries) in order to obtain a healthier meat product with a lower caloric value and a better lipid profile.

## 2. Materials and Methods

### 2.1. Gelled Emulsion Elaboration

#### 2.1.1. Materials

To create the gelled emulsions, walnut oil (61.21% linoleic acid, 14.52% oleic acid, 12.43% linolenic acid, and 6.85% palmitic acid) purchased from Cooks&Co (Isleworth, UK), cocoa bean shell flour (total dietary fibre 61.18 g/100 g; protein content 17.13 g/100 g; catechin and epicatechin content of 4.56 and 6.33 mg/g, respectively; theobromine and caffeine content of 12.27 and 6.13 mg/g, respectively) supplied by chocolates Valor (Villajoyosa, Spain), and gelatin of animal origin (pork) with 180 bloom that was obtained from Sosa Ingredients S.L. (Barcelona, Spain) were used as ingredients.

#### 2.1.2. Elaboration of Oil-in-Water-Gelled Emulsion

**Figure 1:**
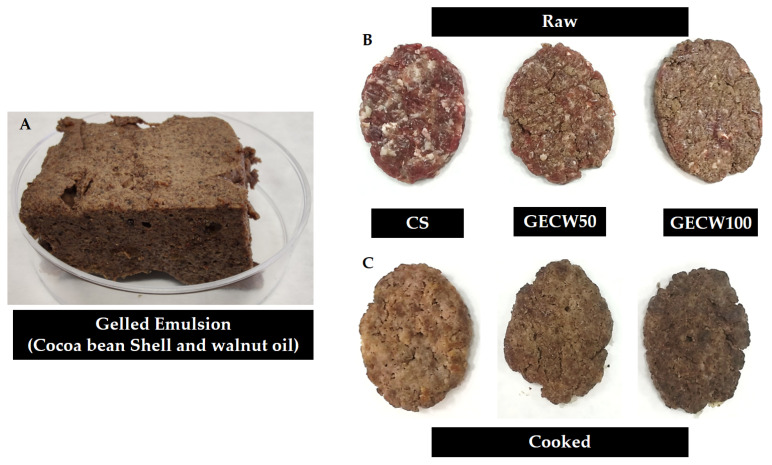
Beef burger made with a gelled emulsion as partial or total pork backfat replacer. (**A**): Gelled emulsion created with cocoa bean shell flour and walnut oil (GECW). (**B**): Raw burger made with 0% (CS: Control sample), 50%, and 100% GECW as fat replacer. (**C**): Cooked burger made with 0%, 50%, and 100% GECW as fat replacer.

Gelled emulsions were prepared at room temperature by mixing the gelling agent 5% (*w*/*v*) with water (40%) at 12,000 rpm for 2 min using a homogenizer Ultra-Turrax, (IKA, Staufen, Germany). Then, this mixture was transferred to a food processor (Thermomix^®^, Vorwerk, Germany), and the cocoa bean shell flour (15% *w*/*v*) was added whilst the food processor was run at 1300 rpm for 2 min. Finally, the walnut oil (40% *w*/*v*) was gradually added into the mixture. After that, the emulsification process was finished by further mixing at 5000 rpm for 5 min. The emulsion obtained was cold-set at 2 °C for 5 h to ensure gel formation (Figure 1A).

### 2.2. Preparation and Reformulation of Beef Burgers

Beef (*Semimembranosus*) with 74.43% moisture, 2.37% fat, 22.53% protein, and 0.67% ash and pork backfat with 10.59% moisture, 74.41% lipids, 14.53% protein, and 0.47% ash at 48 h post mortem were obtained from a local butchery supplier (Orihuela, Spain). To create the beef burger, a traditional formula was used. This original formula was used as a control sample (CS), whereas the other two formulations, where different proportions of fat (50 or 100%) were replaced by gelled emulsion created with cocoa bean shell flour and walnut oil (GECW), were fabricated as shown in Table 1.

To obtain the control sample, beef meat and pork backfat were cut into approximately 5 cm cubes and were ground using an 8 mm plate in a mincer attached to a mixer. After that, water, salt, and pepper were added into the bowl and were mixed with a spiral dough hook at medium speed (80 rpm) for 4 min. Raw burgers (80 g) were produced using a conventional burger-maker with the dimensions of a 9 cm diameter and 1 cm of thickness. Plastic packaging films were used to preserve the shape of the beef burger before it was packed into PVC-lined hermetic boxes and stored at 4 °C (Figure 1B).

Beef burgers (five from each formulation) were cooked according to the methodology reported by the American Meat Science Association [16] at 170 °C in a convection oven until an internal temperature of 72 °C was obtained, which was taken in the geometrical centre of each patty with a thermocouple (Figure 1C).

### 2.3. Chemical Composition of Beef Burgers

The total moisture, protein, fat, and ash contents of the raw and cooked burgers were determined according to the guidelines of the Association of Official Analytical Chemists [17].

### 2.4. Fatty Acid Profile and Health Indices of Beef Burgers

#### 2.4.1. Fatty Acid Profile

For the analysis of the fatty acid profile, raw and cooked burger were subjected for fat extraction following the method described by Folch et al. [18]. Then, all samples were transmethylated following the recommendations of Golay and Moulin [19]. Fatty acid methyl esters (FAMEs) were analysed in gas chromatography equipment Hewlett-Packard 6890 with a flame ionization detector (FID) and a Suprewax 280 capillary column (30 m, 0.25 μm film thickness 0.25 mm i.d.; Tecknokroma Barcelona, Spain) and was carried out according to the chromatographic conditions described by Pellegrini et al. [20]. Results were expressed as g fatty acid/100 g of fat.

#### 2.4.2. Health Indices

Health indices of the different samples were obtained to assess the nutritional quality of the fat composition from the beef burgers: Total saturated (SFA), monounsaturated (MUFA), and polyunsaturated (PUFA) fatty acids contents and the n-6 and n-3 fatty acid ratio and the PUFA and SFA ratio were obtained. In the same way, the atherogenic index (AI) and thombogenic index (TI) were calculated following Equations (1) and (2), as proposed by Ulbricht and Southgate [21]:

(1) $$\mathrm{IA}=\frac{C12:0+\left(4\mathrm{xC}14:0\right)+C16:0}{\sum^{\;}\mathrm{MUFA}+\sum^{\;}n-6+\sum^{\;}n-3}$$

(2) $$\mathrm{IT}=\frac{C14:0+C16:0+C18:0}{(0.5x\sum^{\;}\mathrm{MUFA})+(0.5x\sum^{\;}n-6)+(3x\sum^{\;}n-3)+\left(\frac{\sum^{\;}n-3}{\sum^{\;}n-6}\right)}$$

The hypocholesterolemic and hypercholesterolemic ratio was calculated with Equation (3), as described Fernández et al. [22]:

(3) $$\frac{h}{H}=\frac{C18:1n-9+C18:1n-7+\sum^{\;}\mathrm{PUFA}}{C14:0+C16:0}$$

### 2.5. Physic-Chemical Properties of Beef Burgers

#### 2.5.1. Colour Parameters

The colour properties of the raw and cooked burger were studied in the CIEL *a* b* colour space using a Minolta CM-700 (Minolta Camera Co., Osaka, Japan) with illuminant D65, Specular Component Included (SCI) mode and an observer angle of 10°. Low reflectance glass (Minolta CR-A51/1829-752) was placed between the samples and the equipment. Guidelines for meat colour measurements from the American Meat Science Association described in Hunt et al. [23] and the recommendations of Sanchez-Zapata et al. [24] were used to determine the infinite solid (product thickness) and background. The CIEL *a* b* coordinates that were determined were lightness (L*), redness (a*, coordinate red/green), and yellowness (b*, coordinate yellow-blue), and the psychophysical parameters that were determined were h * ab (hue) and C* (chroma), which were calculated as follows:

(4) $$C{*}^{\;}=\sqrt{a^{*2}+b^{*2}}$$

(5) $${H^{*}}_{\mathrm{ab}}=\mathrm{arctang}(\frac{b^{*}}{a^{*}})$$

The total colour differences (ΔE) of each sample (S) with respect to the control beef burger (C) were also calculated as follows:

(6) $$\Delta E=\sqrt{{\left(L_{S}^{*}-L_{C}^{*}\right)}^{2}+{\left(a_{S}^{*}-a_{C}^{*}\right)}^{2}+{\left(b_{S}^{*}-b_{C}^{*}\right)}^{2}}$$

#### 2.5.2. pH

The pH of the raw and cooked burgers was measured using a penetration probe at different sites of the sample with a pH-meter Crison model 510, (Barcelona, Spain).

#### 2.5.3. Texture

Texture profile analysis-TPA was performed in cooked beef samples using a TA-XT2i Texture Analyzer (Stable Micro Systems, Surrey, England). Cubic samples of 1 cm^3^ were submitted to two compression cycles. Samples were compressed to 75% of their original height with a cylindrical probe of 10 cm diameter at a compression load of 25 kg with a constant velocity of 1 mm/s at 15–20 °C. From the curves that were obtained (force-time deformation), the following parameters were calculated: hardness (N), springiness (mm), cohesiveness, and chewiness (N *mm) [25].

### 2.6. Cooking Characteristics of Beef Burgers

The weight, thickness, and diameter of the beef burgers from each batch were measured at room temperature before and after cooking. To estimate the dimensional changes, the reductions in the diameter and the thickness increases were calculated from the following equations:

The diameter reduction was calculated according to Equation (7); the thickness increase was calculated according to Equation (8); and to estimate the cooking loss, Equation (9) was performed:

(7) $$\mathrm{Shrinkage}\;\left(\%\right)=\frac{\left(\mathrm{raw}\;\mathrm{diameter}-\mathrm{cooked}\;\mathrm{diameter}\right)}{\left(\mathrm{raw}\;\mathrm{diameter}\right)}x100$$

(8) $$\mathrm{Thickness}\;\mathrm{increase}\;\left(\%\right)=\frac{\left(\mathrm{Cooked}\;\mathrm{thickness}-\mathrm{raw}\;\mathrm{thickness}\right)}{\left(\mathrm{Raw}\;\mathrm{thickness}\right)}x100$$

(9) $$\%\;\mathrm{Cooking}\;\mathrm{loss}=\frac{\left(\mathrm{Raw}\;\mathrm{weight}-\mathrm{Cooked}\;\mathrm{weight}\right)}{\left(\mathrm{Raw}\;\mathrm{weight}\right)}\;x\;100\;$$

### 2.7. Polyphenolic Profile of Beef Burgers

#### 2.7.1. Extract Preparation

The extraction of the (poly)phenolic and methylxanthine compounds from the beef burgers was divided into two fractions: (*i*) extracts with the free (poly)phenolic compounds and (*ii*) extracts with bound-insoluble (poly)phenolic compounds. To obtain the free (poly)phenolic extracts, the methodology reported by Genskowsky et al. [26] was used. The bound-insoluble (poly)phenolic compounds were obtained using the methodology described by Mpofu et al. [27] and by using the pellet that remained after the extraction of the free polyphenolic compounds. Finally, to avoid any molecule interfering with the chromatographic analyses, the extracts were loaded onto a C-18 Sep-Pak cartridge that had been previously conditioned. The extracts that were obtained were maintained at −40 °C until high-performance liquid chromatography analysis (HPLC) analysis could be conducted.

#### 2.7.2. High-Performance Liquid Chromatography Analysis

Polyphenolic profiles of the free and bound extracts obtained from all of the cooked samples were determined by high-performance liquid chromatography following the methodology proposed by Genskowsky et al. [26]. The identified compounds were quantified according to the peak area measurements, which were reported in the calibration curves of the corresponding authentic standards.

### 2.8. Methylxanthines Analysis

Theobromine and caffeine contents were determined for the extracts obtained in Section 2.7.1. The HPLC analysis was completed following the methodology described by Grillo et al. [28]. The quantification of caffeine and theobromine were quantified according to the peak area measurements, which were reported in the calibration curves of the corresponding authentic standards.

### 2.9. Lipid Oxidation of Beef Burgers

The methodology describes by Rosmini et al. [29] was applied to determine the 2-thiobarbituric acid reactive substance (TBARs) value for the detection of the lipid oxidation rate of the raw and cooked beef burgers.

### 2.10. Sensory Analysis of Beef Burgers

Forty non-experienced panelists (staff and students from the Miguel Hernández University) comprising 15 males and 25 females who were between 20 and 60 years of age and who had not undergone specific training for sensory burgers analysis were recruited. The sensory analysis protocols were approved by the Project Evaluation Office of the Miguel Hernández University. All of the sensory assays were carried out in the sensory laboratory at the University. For these assays, five beef burgers from each formulation (each sample was coded with randomly selected 3-digit numbers) were cooked as previously described and were maintained warm in an oven until testing, which occurred within 4–7 min. after cooking. Square pieces approximately 1.5 × 1.5 cm were cut from the burger and were served at room temperature. Unsalted crackers and mineral water (room temperature) were provided to clean the palate between samples. Each panelist evaluated all of the formulas in a randomized order and was asked to assign a numerical value between 1 and 9 for the following attributes: colour, bitter taste, cocoa flavour, fat sensation, hardness, and juiciness of the beef burger as well as the general acceptability, where 1 represented dislike extremely and 9 represented like extremely.

### 2.11. Statistical Analysis

The full process (gelled emulsion elaboration and burger manufacture) was replicated three times (three independent batches). Each replication was done on a different production day, and each batch was analysed in triplicate. The results in the tables were expressed as mean values and standard deviations. The data obtained for all of the assays were analysed by means of a one-way Analysis of Variance (ANOVA) test, and Tukey’s post hoc test was applied for the comparison of the means; differences were considered significant at *p* < 0.05. All statistics assays were performed using the statically package SPSS v. 27 for windows (SPSS INC., Chicago, IL, USA).

## 3. Results

### 3.1. Chemical Composition

The chemical compositions of the raw and cooked beef burger that had been elaborated with cocoa bean shell flour and walnut oil emulsion gel as partial and total pork backfat replacers are shown in Table 2. In the raw and cooked samples, both the partial and total substitution of pork backfat with the gelled emulsion had a deep impact on all of the parameters that were analysed. Thus, the moisture and ash content increased in the GECW50 and GEWC100 samples (*p* < 0.05) with respect to CS. However, the protein and fat content decreased (*p* < 0.05) in the GECW50 and GEWC100 samples compared to CS and did so in a manner that was dependent on the degree of substitution. The increasing moisture content could be explained by the replacement of the pork backfat, which had a moisture content of 10.59 g/100 g, by the gelled emulsion, which had a water content of 40 g/100 g.

These results were expected and were in agreement with the results obtained by Barros et al. [9], who replaced pork backfat with algal and wheat germ oil emulsions in beef burgers, or Serdaroğlu et al. [30], who used a gelled emulsion prepared with olive oil as a beef fat replacer in chicken burgers. The reduction in protein and fat content can be attributed to the fact that the pork backfat that showed a fat and protein content of 74.41 and 14.53 g/100 g, respectively, was substituted by a gelled emulsion that only contained 40 g/100 g of oil and 7.20 g/100 g of proteins. The same behaviour was observed by Alejandre et al. [31] in meat batters using canola oil hydrogels as a fat replacer and by Lucas-González et al. [32] in pork burgers where the pork backfat was replaced by gelled emulsions elaborated with chia oil and chestnut flour. Finally, the ash content increased as the pork backfat decreased in the burger formulations. This could be explained by the presence of cocoa bean shell flour in the emulsion. It is important to note that the burgers obtained in this work, where the pork fat was replaced by a gelled emulsion, could be considered to have a lower nutritional value since the protein content decreases and the fat content increases once they are cooked. However, it should not be forgotten that the replacement of saturated fatty acids with polyunsaturated acids could compensate for this nutritional loss due to the reduction in the protein content.

### 3.2. Fatty Acid Profile and Health Indices

The fatty acid profiles of the raw and cooked beef burgers elaborated with cocoa bean shell flour and walnut oil emulsion gels as partial and total pork backfat replacers are given in Table 3. The beef burger reformulation significantly enhanced the fatty acid profile. In the raw burgers, the CS showed a higher content (*p* < 0.05) of saturated fatty acids, mainly stearic (C16:0) and palmitic (C18:0) acid, as well as a higher content of the monounsaturated fatty acid (*p* < 0.05) oleic acid (C10:1n9), which was the predominant component that was compared with the reformulated burgers. Since walnut oil was used in the gelled emulsion formulation, the lipid profile of this oil was reproduced in the GECW50 and GEWC100 samples, which explains the rising concentrations of linoleic acid (C18:2n6) from 9.67% in CS to 28.65% in GECW50 and 40.42% in GECW100 as well as in the linolenic acid (C18:3n3) in CS, which increased from 0.62% to 5.40% in GECW50 and to 8.48 % in GECW100. It is important to notice that due to the reformulation of the burgers, the total saturated fatty acids decreased (*p* < 0.05) from 34.38% in CS to 24.75% in GECW100 due to the significantly lower values of stearic and palmitic acids obtained in the reformulated samples whilst the monounsaturated fatty acids fells (*p* < 0.05) from 54.17% in CS to 25.77% in GECW100 due to the reduction in oleic acid observed in reformulated samples. On the other hand, a high increase was obtained in the content of polyunsaturated fatty acids, which augmented (*p* < 0.05) from 11.38% in CS to 49.02% in GECW100. This behaviour is consistent with the scientific literature, where it has been that the replacement of animal fat by gelled emulsions elaborated with healthy oil provokes a significant reduction in the saturated and monounsaturated fatty acid fractions and a very high increase in the polyunsaturated fatty acid fraction [33,34,35]. In cooked burger, the same trend was observed: the saturated and monounsaturated fatty acids fell (*p* < 0.05) from 38.85% in CS to 23.90% in GECW100 and from 50.75% in CS to 25.01% in GECW100, while the polyunsaturated fatty acids increased (*p* < 0.05) from 10.35% in CS to 50.63% in GECW100.

Regarding the health indices (Table 3), in the raw burgers, both the partial and total pork backfat replacement of animal fat by gelled emulsion elaborated with cocoa bean shell flour and walnut oil had a significant effect (*p* < 0.05) on ΣPUFA/ΣSFA and the Σn6/Σn3 and the hypocholesterolemic/hypercholesterolemic ratios as well as on the atherogenicity and thrombogenicity indices. The ΣPUFA/ΣSFA ratio in the GECW50 and GECW100 burgers was 1.33 and 1.98, respectively, while in CS, it was 0.33. In this sense, Heck et al. [36] indicated that the recommended ΣPUFA/ΣSFA ratio should be higher than 0.4. Therefore, the results obtained in the reformulated samples showed an optimal ΣPUFA/ΣSFA. Similarly, the hypocholesterolemic/hypercholesterolemic ratio increased in the reformulated samples with respect to CS. As mentioned by Barros et al. [9], the high values of the hypocholesterolemic/hypercholesterolemic ratio indicate that the meat product is healthier than products with lower ratio levels. As may be expected from the Σn6/Σn3 ratio in animal fat, the values obtained in CS were higher (10.48) than those obtained in the GECW50 and GECW100 samples, which had Σn6/Σn3 ratios of 5.05 and 4.07, respectively. It is important to note that according to the nutritional recommendations of the Food and Agriculture Organization of the United Nations [37], the Σn6/Σn3 ratio should be less than 4.0.

The healthy meat products should have atherogenicity and thrombogenicity value indices that are as low as possible. The atherogenicity index decreased by about 33.32% and 40.47% in GECW50 and GECW100, respectively, with respect to CS, while the thrombogenicity index fell to about 46.8% and 57.45% in GECW50 and GECW100 with respect to CS. Food products in general and meat products in particular that have low values for the atherogenicity and thrombogenicity indices may inhibit platelet aggregation and decrease the levels of esterified fatty acids, cholesterol, and phospholipids and may consequently reduce the risk of developing cardiovascular diseases [21]. In cooked burgers (Table 3), the same behaviour was observed. The atherogenicity and thrombogenicity indices in reformulated samples decreased with respect to CS as well as the Σn6/Σn3, ratio whereas the ΣPUFA/ΣSFA and the hypocholesterolemic/hypercholesterolemic ratios increased in the GECW50 and GECW100 samples with respect to CS.

These results were consistent with the results reported by several authors who mentioned that beef or pork burgers where the animal fat was replaced, either partially or totally, by gelled emulsion elaborated with healthy oils showed better health indices compared to samples formulated with animal fat [9,32,34,36].

### 3.3. Physic-Chemical Properties

The physic-chemical properties of meat products in general and burgers in particular can be altered by the type and concentration of different lipid systems, such as gelled emulsions, being used in the reformulation, which can change the conventional appearance and textural properties of this type of product. Table 4 shows the physic-chemical properties of raw and cooked beef burger elaborated with cocoa bean shell flour and walnut oil emulsion gel as partial and total pork backfat replacers. The pH values of the raw and cooked GECW50 and GECW100 burgers were significantly (*p* < 0.05) lower than that of the CS. The increase in the replacement levels of the gelled emulsion was related to a reduction of the pH values. These results agree with those reported by Lu et al. [38], where comparable trends in pH changes were reported for pork patties formulated with a 40% fat replacement using olive oil, sunflower oil, or grape seed oil.

Colour is one of the most important aspects of consumer acceptance toward meat and meat products. For the raw and cooked burgers with the partial and total replacement of pork backfat with a gelled emulsion elaborated with cocoa bean shell flour and walnut oil, differences in lightness (L*), redness (a*), and yellowness (b*) and the psychophysical parameters hue (h*) and chroma (C*) were statistically significant (*p* < 0.05) when compared to the control sample (Table 4). Thus, the raw CS had lower values of L*, b*, h*, and C* than the GECW50 and GECW100 burgers. However, when the burgers were cooked, it was the CS that presented higher values of L*, b*, h*, and C* than those found in the GECW50 and GECW100 burgers. On the other hand, for both the raw and cooked burgers, no statistical differences (*p* > 0.05) were found in terms of the redness (a*) between all of the samples. In the scientific literature, controversial results have been reported regarding the effect of fat substitution by gelled emulsions in pork or beef burgers in terms of colour parameters [39,40,41]. Several factors could be responsible for this, such as the colour of the oil, the characteristics and composition of the oil, and the colour of the emulsifiers or gel-ling agents as well as the interaction of all of the ingredients between them. Another important parameter to consider when evaluating colour modifications as results of meat product reformulation is the colour differences (ΔE*) with respect to the original sample (control). Several works have mentioned that values of over 3 units correspond to changes that are perceptible by the human eye [42]. Table 4 shows the colour differences for the raw and cooked beef burgers where the pork backfat was partially or totally replaced by a gelled emulsion. For both the raw and cooked GECW50 and GECW100 burgers, the colour clearly differed from that of the CS. These colour differences could affect negatively consumer acceptance because these differences are classified as being medium discordant [43].

**Figure 2:**
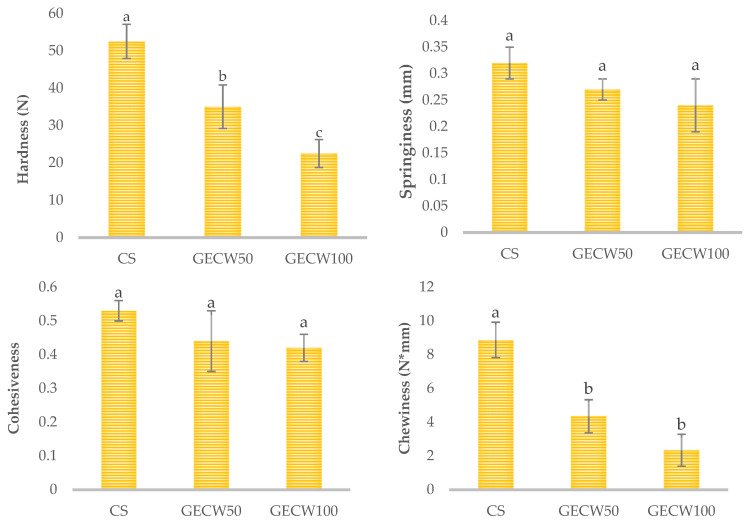
Textural profile analysis of cooked beef burger elaborated with cocoa bean shell flour and walnut oil emulsion gel as partial and total pork backfat replacers. CS: control sample elaborated with the traditional formula; GECW50: sample with 50% gelled emulsion elaborated with cocoa bean shell flour and walnut oil as fat replacer; GECW100: sample with 100% gelled emulsion elaborated with cocoa bean shell flour and walnut oil as fat replacer. Bars with different letters are significantly different (*p* < 0.05).

The results of the texture profile analysis (TPA) of the cooked beef burger elaborated with cocoa bean shell flour and walnut oil emulsion gel as partial and total pork backfat replacers are shown in Figure 2.

For hardness, as the degree of fat substitution increased, there was a decrease (*p* < 0.05) in the values obtained for this parameter. This behaviour is in accordance with the values reported by Serdaroğlu et al. [30] in chicken patties where beef fat was replaced with a gelled emulsion prepared with olive oil. These changes in the hardness values of the burgers with partial or total pork backfat substitution may be attributed to two aspects as mentioned Freire et al. [44]. On the one hand, the chemical characteristics of the meat matrix formed and showed differences in protein, water (high), and fat (low) ratio. On the other hand, there were differences in the physic-chemical properties of the pork backfat and gelled emulsion. For springiness and cohesiveness, no statistical differences (*p* > 0.05) were found between CS and GECW50 and GECW100. These results were in accordance with those reported by Cittadini et al. [33] in foal burgers where the animal fat was replaced by oil mixture emulsion hydrogels. In the case of chewiness, which showed similar trends as the hardness, statistical differences (*p* < 0.05) were obtained between the CS and GECW50 or GECW100 samples, which did not show statistical (*p* > 0.05) differences. This tendency is in contrast to the results of several authors, who observed an increase in the chewiness values of burgers where the pork backfat was substituted by gels containing pork skin and canola oil [45] or in burgers that used a hydrogelled emulsion using chia and linseed oils as fat replacers [36].

### 3.4. Physic-Chemical Properties

The cooking characteristics, including cooking loss, shrinkage, or thickness increases, are some of the most significant issues for the food industry when predicting the behaviour of several meat products during the cooking process [30]. The cooking characteristics of beef burgers elaborated with cocoa bean shell flour and walnut oil emulsion gel as partial and total pork backfat replacers are given in Table 5. In terms of cooking loss, GECW100 had a lower cooking loss (*p* < 0.05) compared to the other formulations, with the CS showing the highest values. This phenomenon may be explained by the fact that in GECW100, several components of cocoa bean shell flour, such as dietary fibre, could produce hydrogen bonds with water and could retain the moisture in the meat matrix. In the same way, interactions among water, cocoa bean shell flour components, and gelatin may also occur. This reduction in cooking loss is in agreement with the values reported by Barros et al. [46] in beef burgers with a tiger nut oil emulsion as fat substitute.

Cooking losses are affected by several factors, including the cooking method, final cooking temperature, the pH of the sample, and amount and type of fat [47,48]. In reference to shrinkage (Table 5), CS had the highest (*p* < 0.05) values, whilst no statistical differences (*p* > 0.05) were found between GECW50 and GEWC100. Cooking shrinkage, which occurs because of protein denaturation that causes a fat and water release from the beef burger, is influenced by the reduction in the fat content. Thus, a reduction in the amount of fat led to a reduction in burger shrinkage, as reported Serdaroğlu and Degirmencioglu [49]. The values obtained in this work are supported by the results obtained by Barros et al. [9], who determined that gelled emulsions elaborated with algal oil and wheat germ utilized as fat substitute in beef burgers similarly reduced shrinkage during cooking. Regarding increases in the thickness, once again, the control sample showed the highest value (*p* < 0.05), while GECW50 and GEWC100 had a reduction in the thickness increase (*p* < 0.05), and this may have been dependent on the degree of fat replacement.

### 3.5. Polyphenol and Methylxanthines Content

**Figure 3:**
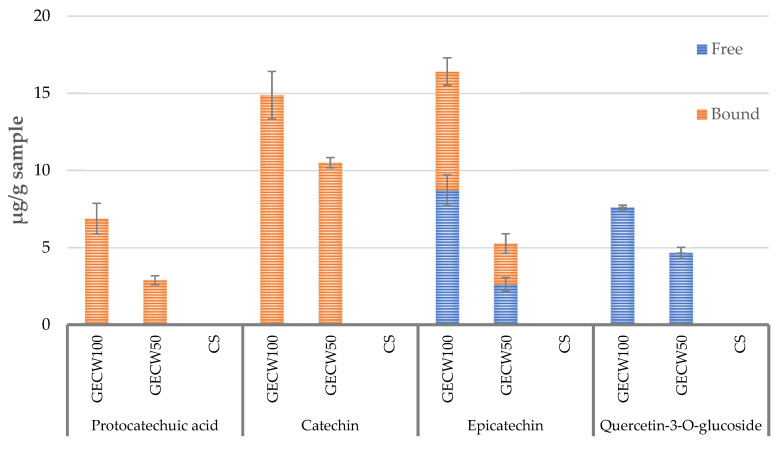
Bound and free (poly)phenol compounds identified in cooked beef burger elaborated with cocoa bean shell flour and walnut oil emulsion gel as partial and total pork backfat replacers. CS: control sample elaborated with the traditional formula; GECW50: sample with 50% gelled emulsion elaborated with cocoa bean shell flour and walnut oil as fat replacer; GECW100: sample with 100% gelled emulsion elaborated with cocoa bean shell flour and walnut oil as fat replacer.

**Figure 4:**
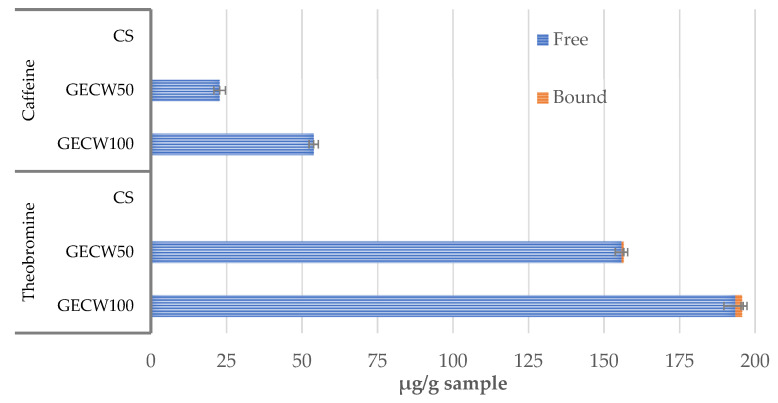
Bound and free methylxanthines identified in cooked beef burger elaborated with cocoa bean shell flour and walnut oil emulsion gel as partial and total pork backfat replacers. CS: control sample elaborated with the traditional formula; GECW50: sample with 50% gelled emulsion elaborated with cocoa bean shell flour and walnut oil as fat replacer; GECW100: sample with 100% gelled emulsion elaborated with cocoa bean shell flour and walnut oil as fat replacer.

In this work, the bound and free polyphenol content (Figure 3) and bound and free methylxanthine content (Figure 4) of cooked beef burgers elaborated with cocoa bean shell flour and walnut oil emulsion gel as partial and total pork backfat replacers were determined. In terms of the polyphenol content (Figure 3), in both the reformulated burgers (GECW50 and GEWC100), three bound polyphenols and two free polyphenols, which were provided by the cocoa bean shell flour since those compounds were not detected in CS, were identified. Regarding the bound fraction, in both GECW50 and GEWC100, the flavan-3-ols catechin and epicatechin were the predominant (*p* < 0.05) compounds followed by protocatechuic acid in a lower concentration, which were the expected results. These results were in concordance with the data reported in the literature by Hernández-Hernández et al. [14] and by Jokíc et al. [50], who reported that epicatechin and catechin are the main polyphenolic compounds that are in cocoa bean shell. In reference to the free fraction, only epicatechin and quercetin-3-*O*-glucoside were detected. It is important to note that catechin was not detected in the free fraction, as this compound is one of the main polyphenolic compounds that is present in cocoa bean shell [51]. In the same sense, the rest of the (poly)phenolic compounds present in cocoa bean shell flour were gradually lost during the elaboration process.

In the case of methylxanthines (Figure 4), in both the GECW50 and GEWC100 burgers, only theobromine was detected, but it was detected at a very low concentration (0.69 and 2.19 µg/g sample for GECW50 and GEWC100, respectively,) in the bound fraction, while caffeine and theobromine were detected in the free fraction. The increase in the levels of the gelled emulsion, which was used as fat replacer, was related to the increase of the methylxanthine content (*p* < 0.05). Thus, the GECW100 sample showed a theobromine and caffeine content of 193.50 and 53.80 µg/g, respectively, while in GECW50 sample, the theobromine and caffeine content were 155.80 and 22.73 µg/g, respectively. The identification of these compounds can be expected since theobromine and caffeine are the major phytochemicals found in cocoa bean shell [15]. These compounds have been described to exert several physiological effects in the human body, including in the nervous, respiratory, and cardiac systems [52].

### 3.6. Lipid Oxidation

**Figure 5:**
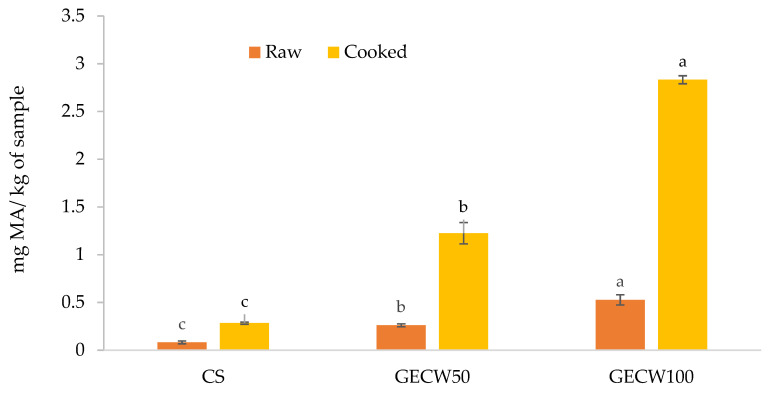
Lipid oxidation values of raw and cooked beef burger elaborated with cocoa bean shell flour and walnut oil emulsion gel as partial and total pork backfat replacers. CS: control sample elaborated with the traditional formula; GECW50: sample with 50% gelled emulsion elaborated with cocoa bean shell flour and walnut oil as fat replacer; GECW100: sample with 100% gelled emulsion elaborated with cocoa bean shell flour and walnut oil as fat replacer. For raw or cooked samples bars with the same letter are not significantly different (*p* > 0.05) according to Tukey’s multiple range test.

Lipid oxidation along with microbial spoilage are the two main causes of sensory deterioration and shelf-life reduction in fresh meat products such as burgers. The oxidative stability of beef burger elaborated with cocoa bean shell flour and walnut oil emulsion gel as partial and total pork backfat replacers was assessed by measuring the malonaldehyde content (Figure 5), which is the principal secondary product resulting from the decomposition of polyunsaturated fatty acid hydroperoxides [53]. In the raw burgers, GECW100 showed higher (*p* < 0.05) TBARs values (0.53 mg malonaldehyde/kg sample) than GECW50 and CS. In cooked burger, thermal treatment provoked the lipid oxidation values to increase. As is well known, the oxidation process is strongly improved when meat products are cooked due to the thermal treatment accelerating oxidative reactions in the meat products. Again, GECW100 showed higher (*p* < 0.05) TBARs values. However, it is important to highlight that the CS and GECW, with the exception of cooked GECW100, showed values below the threshold limit that could be perceived by consumers (2 mg malonaldehyde/kg sample), which could be considered the maximum acceptable value [54].

This increase in the lipid oxidation values of the reformulated beef burgers was expectable due to the elevated content in the polyunsaturated fatty acids of the gelled emulsions that were used as fat replacers. This fact is in agreement with several works [32,36,55] that have reported that the use of vegetable and marine oils, as functional ingredients in the development of lipid emulsions for animal fat replacement, could be complicated due to the high oxidation susceptibility of these matrices due to the high content of the polyunsaturated fatty acids of these oils. On the other hand, some works [38,46] have reported that gelled emulsions elaborated with vegetable oils or flours, where it is possible to find several compounds with antioxidant activity, including tocopherols or polyphenolic compounds, in their composition, the TBARs values decreased with respect to the control sample. In this work, this is not the case despite the fact that the GECW50 and GECW100 samples showed the presence of antioxidant compounds, including the polyphenols (catechin and epicatechin) and methylxanthines (theobromine and caffeine) from the cocoa bean shell flour. This may be due, in part, to the low concentration in which they were found in the final product; as is well known, the better or worse oxidative stability of meat products depends on an adequate balance between antioxidant and prooxidant compounds. On the other hand, these compounds were found in a higher concentration in the bound fraction, which was probably due to the dietary fibre content, which means that they are not able to exert this antioxidant activity.

### 3.7. Sensorial Analysis

Sensory analysis is particularly essential because it indicates product quality as well as consumer acceptance. In addition, when different strategies are used to reduce the fat content of meat products, their sensory analysis must be taken into account since fat plays a very important role in this type of product from a sensory point of view. The fat that is present in meat products has positive effects on texture, juiciness, colour, tenderness, and overall palatability as well as on the formation of characteristic and desirable aromas and flavour (lipid-derived volatiles, lipolysis, moderate lipid oxidation, etc.) of these products [6].

**Figure 6:**
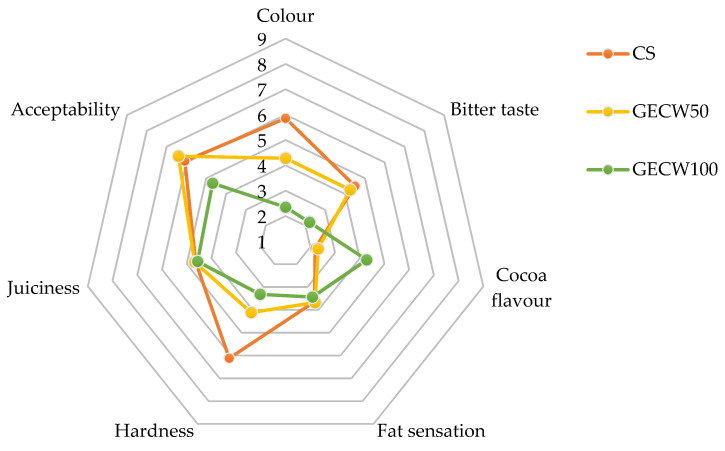
Sensory properties of cooked beef burger elaborated with cocoa bean shell flour and walnut oil emulsion gel as partial and total pork backfat replacers. CS: control sample elaborated with the traditional formula; GECW50: sample with 50% gelled emulsion elaborated with cocoa bean shell flour and walnut oil as fat replacer; GECW100: sample with 100% gelled emulsion elaborated with cocoa bean shell flour and walnut oil as fat replacer.

The sensory properties of cooked beef burger elaborated with cocoa bean shell flour and walnut oil emulsion gel as partial and total pork backfat replacers are exhibited in Figure 6. The colour score of the CS was significantly higher (*p* < 0.05) than that of GECW50 and GECW100. These results were in agreement with instrumental analysis, which detected several changes in the colour parameters between the CS and the burgers where the fat was replaced. The panelists evaluated the GECW50 and GECW100 samples with the highest scores for bitter taste (*p* < 0.05), which was valued in a positive way. Regarding cocoa flavour, GECW100 had the highest (*p* < 0.05) scores, while no statistical differences were found (*p* > 0.05) between CS and GECW50. This result was expected since the cocoa bean shell used to make the gelled emulsion stood out notably. For fat sensation, no differences were found between the CS and the burgers where the fat was replaced. This result is in contrast to the fat values measured in an instrumental way. Therefore, the panelists showed that there were no differences between the samples even though instrumentally significant differences were found among them. For hardness, as was also the case with colour, the panelists scored the CS higher (*p* < 0.05) than the burgers with partial and total pork backfat substitution values, which was in agreement with the values obtained in the textural assay that showed that CS had higher values for this parameter than GECW50 and GECW100 did. For juiciness, no statistical differences (*p* > 0.05) were found between the samples. Finally, for acceptability, the lowest scores (*p* < 0.05) were achieved for GECW100, whilst the most acceptable samples were CS and GECW50, with no statistical differences (*p* < 0.05) being determined among the samples.

## 4. Conclusions

Co-products from agri-food industries (cocoa processing) may be novel sources of emulsifiers for structuring vegetal oils (walnut oil) as gelled emulsions, making them applicable for use as fat replacers in frequently consumed meat products such as beef burgers. These co-products (depending on their origin) could be interesting sources of bioactive compounds (polyphenols, carotenoids, methylxanthines, etc.) with functional properties. Healthier burgers (a better lipid profile according to recommendations for healthy fats) without technological problems have been successfully obtained using a gelled emulsion (based on cocoa co-products and walnut oil) as a pork back fat replacer (even when reaching total pork backfat replacement). This novel reformulation strategy also affects lipid oxidation development (mainly after cooking) and certain sensory properties (such as colour and flavour). Although this reformulation process entailed changes in sensory characteristics, when this gelled emulsion was only used as partial replacement (50%) for animal fat in burgers, the burgers were judged to be acceptable and did not show any differences with the control burgers.

These interesting results could contribute not only to providing healthier meat products to the population but also to the valorisation of co-products from agri-food industries, contributing to the sustainability of food industries sustainability as well as to the circular economy.

## Figures and Tables

**Table 1:** Formulation of beef burgers with cocoa bean shell flour and walnut oil emulsion gel used as partial (50%) and total pork backfat replacers.

	Treatments (%)		
	CS	GECW50	GECW100
Beef	70	70	70
Pork backfat	30	15	0
Water	5	5	5
Salt	1.5	1.5	1.5
White pepper	0.05	0.05	0.05
GECW	0	15	30

Percentages of non-meat ingredients are related to 100% meat. CS: control sample. GECW: gelled emulsion elaborated with cocoa bean shell flour and walnut oil.

**Table 2:** Chemical composition of the raw and cooked beef burger elaborated with cocoa bean shell flour and walnut oil emulsion gel as partial and total pork backfat replacers.

	Raw			Cooked		
	CS	GECW50	GECW100	CS	GECW50	GECW100
Moisture	61.90 ± 0.14 ^c^	66.76 ± 0.09 ^b^	68.20 ± 0.09 ^a^	55.60 ± 0.31 ^c^	57.14 ± 0.06 ^b^	60.01 ± 0.08 ^a^
Protein	19.75 ± 1.41 ^a^	17.92 ± 1.09 ^b^	14.16 ± 1.36 ^c^	26.11 ± 0.15 ^a^	23.16 ± 0.39 ^b^	19.90 ± 0.50 ^c^
Fat	15.93 ± 0.09 ^a^	12.63 ± 0.24 ^b^	11.38 ± 0.10 ^c^	12.30 ± 0.11 ^b^	16.48 ± 0.11 ^a^	16.31 ± 1.10 ^a^
Ash	1.94 ± 0.04 ^b^	2.21 ± 0.16 ^a^	2.25 ± 0.04 ^a^	2.22 ± 0.03 ^b^	2.43 ± 0.05 ^a^	2.50 ± 0.09 ^a^

Values are expressed as g/100 g. CS: control sample elaborated with the traditional formula; GECW50: sample with 50% gelled emulsion elaborated with cocoa bean shell flour and walnut oil as fat replacer; GECW100: sample with 100% gelled emulsion elaborated with cocoa bean shell flour and walnut oil as fat replacer. For each group (raw or cooked), values followed by the same small letter within the same row are not significantly different (*p* > 0.05) according to Tukey’s multiple range test.

**Table 3:** Fatty acid profile of raw and cooked beef burger elaborated with cocoa bean shell flour and walnut oil emulsion gel as partial and total pork backfat replacers.

	Fatty Acid Profile					
	Raw			Cooked		
	CS	GECW50	GECW100	CS	GECW50	GECW100
C10:0	0.06 ± 0.01 ^a^	0.04 ± 0.01 ^a^	0.02 ± 0.00 ^b^	0.06 ± 0.00 ^a^	0.03 ± 0.00 ^b^	0.02 ± 0.00 ^b^
C12:0	0.06 ± 0.01 ^a^	0.04 ± 0.01 ^ab^	0.03 ± 0.00 ^b^	0.06 ± 0.00 ^a^	0.04 ± 0.00 ^b^	0.03 ± 0.00 ^b^
C14:0	1.30 ± 0.11 ^a^	0.91 ± 0.07 ^b^	0.98 ± 0.08 ^b^	1.55 ± 0.06 ^a^	0.92 ± 0.08 ^b^	0.90 ± 0.04 ^b^
C14:1	0.14 ± 0.04 ^ab^	0.12 ± 0.02 ^b^	0.22 ± 0.05 ^a^	0.26 ± 0.02 ^a^	0.15 ± 0.04 ^b^	0.19 ± 0.02 ^b^
C15:0	0.09 ± 0.02 ^b^	0.09 ± 0.01 ^b^	0.19 ± 0.03 ^a^	0.17 ± 0.01 ^a^	0.11 ± 0.02 ^b^	0.18 ± 0.01 ^a^
C15:1	0.14 ± 0.03 ^b^	0.22 ± 0.03 ^a^	0.08 ± 0.01 ^c^	0.30 ± 0.02 ^a^	0.17 ± 0.01 ^b^	0.07 ± 0.00 ^c^
C16:0	22.45 ± 1.25 ^a^	17.01 ± 1.04 ^b^	14.98 ± 1.25	24.22 ± 1.04 ^a^	16.42 ± 0.87 ^b^	14.40 ± 0.63 ^c^
C16:1	3.53 ± 0.49 ^a^	2.18 ± 0.38 ^b^	1.44 ± 0.54 ^c^	3.63 ± 0.84 ^a^	2.18 ± 0.24 ^b^	1.34 ± 0.09 ^c^
C17:0	0.28 ± 0.06 ^ab^	0.24 ± 0.07 ^b^	0.36 ± 0.04 ^a^	0.43 ± 0.01 ^a^	0.26 ± 0.02 ^c^	0.35 ± 0.04 ^b^
C17:1	0.32 ± 0.07 ^a^	0.22 ± 0.05 ^b^	0.22 ± 0.03 ^b^	0.38 ± 0.05 ^a^	0.23 ± 0.04 ^b^	0.23 ± 0.04 ^b^
C18:0	9.77 ± 0.96 ^a^	7.92 ± 0.87 ^b^	7.93 ± 0.87 ^b^	11.95 ± 0.94 ^a^	7.63 ± 0.97 ^c^	7.75 ± 0.98 ^b^
C18:1n-9	48.99 ± 1.79 ^a^	34.69 ± 1.42 ^b^	23.48 ± 1.22 ^c^	45.35 ± 2.07 ^a^	33.42 ± 1.19 ^b^	22.89 ± 1.20 ^c^
C18:2n-6	9.67 ± 1.08 ^c^	28.65 ± 1.37 ^b^	40.42 ± 2.04 ^a^	8.42 ± 1.28 ^c^	30.55 ± 1.04 ^b^	41.46 ± 2.08 ^a^
C18:3n-3	0.62 ± 0.02 ^c^	5.40 ± 0.89 ^b^	8.48 ± 0.68 ^a^	0.77 ± 0.03 ^c^	5.82 ± 0.44 ^b^	8.67 ± 0.79 ^a^
C20:0	0.17 ± 0.01 ^a^	0.15 ± 0.04 ^a^	0.12 ± 0.05 ^a^	0.16 ± 0.01 ^a^	0.14 ± 0.04 ^a^	0.12 ± 0.04 ^a^
C20:1	0.88 ± 0.04 ^a^	0.54 ± 0.06 ^b^	0.17 ± 0.03 ^c^	0.66 ± 0.04 ^a^	0.48 ± 0.07 ^b^	0.17 ± 0.03 ^c^
C20:2n-11	0.48 ± 0.03 ^a^	0.25 ± 0.08 ^b^	0.04 ± 0.00 ^c^	0.34 ± 0.04 ^a^	0.23 ± 0.04 ^b^	0.05 ± 0.00 ^c^
C20:3n-8	0.14 ± 0.01 ^a^	0.12 ± 0.03 ^a^	0.08 ± 0.01 ^b^	0.19 ± 0.03 ^a^	0.11 ± 0.02 ^b^	0.10 ± 0.01 ^b^
C20:3n-11	0.38 ± 0.02 ^a^	0.33 ± 0.07 ^a^	0.12 ± 0.02 ^b^	0.54 ± 0.02 ^a^	0.30 ± 0.02 ^b^	0.24 ± 0.04 ^b^
C24:0	0.07 ± 0.01 ^a^	0.08 ± 0.02 ^a^	0.05 ± 0.01 ^a^	0.13 ± 0.02 ^a^	0.07 ± 0.00 ^b^	0.07 ± 0.01 ^b^
ΣSFA	34.38 ± 1.23 ^a^	26.58 ± 1.37 ^b^	24.75 ± 1.80 ^b^	38.85 ± 1.63 ^a^	25.70 ± 1.73 ^b^	23.90 ± 1.85 ^b^
ΣMUFA	54.17 ± 1.57 ^a^	38.07 ± 1.49 ^b^	25.77 ± 1.73 ^c^	50.75 ± 1.42 ^a^	36.76 ± 1.55 ^b^	25.01 ± 1.09 ^c^
ΣPUFA	11.38 ± 0.96 ^c^	35.03 ± 1.11 ^b^	49.02 ± 1.48 ^a^	10.35 ± 0.74 ^c^	37.19 ± 1.89 ^b^	50.63 ± 2.24 ^a^
	**Health Indices**					
	**Raw**			**Cooked**		
	**CS**	**GECW50**	**GECW100**	**CS**	**GECW50**	**GECW100**
ΣPUFA/ΣSFA	0.33 ± 0.08 ^c^	1.33 ± 0.21 ^b^	1.98 ± 0.18 ^a^	0.27 ± 0.07 ^c^	1.44 ± 0.03 ^b^	2.12 ± 0.04 ^a^
Σn-3	0.99 ± 0.06 ^c^	5.78 ± 0.84 ^b^	8.60 ± 0.94 ^a^	1.32 ± 0.09 ^c^	6.15 ± 0.88 ^b^	8.91 ± 0.73 ^a^
Σn-6	10.38 ± 0.57 ^c^	29.20 ± 1.22 ^b^	40.42 ± 1.74 ^a^	9.03 ± 0.78 ^c^	31.00 ± 1.22 ^b^	41.71 ± 1.74 ^a^
Σn-6/Σn-3	10.48 ± 0.34 ^a^	5.05 ± 0.73 ^b^	4.07 ± 0.61 ^c^	6.84 ± 0.88 ^a^	5.04 ± 0.63 ^b^	4.68 ± 0.57 ^b^
h/H	2.54 ± 0.14 ^c^	3.89 ± 0.12 ^b^	4.54 ± 0.75 ^a^	2.16 ± 0.24	4.07 ± 0.83	4.80 ± 0.72 ^a^
AI	0.42 ± 0.04 ^a^	0.28 ± 0.08 ^b^	0.25 ± 0.06 ^b^	0.50 ± 0.07 ^a^	0.27 ± 0.04 ^b^	0.24 ± 0.05 ^b^
TI	0.94 ± 0.07 ^a^	0.50 ± 0.04 ^b^	0.40 ± 0.06 ^b^	1.11 ± 0.04 ^a^	0.47 ± 0.07 ^b^	0.38 ± 0.04 ^b^

Values are expressed as g/100 g fat. CS: control sample elaborated with the traditional formula; GECW50: sample with 50% gelled emulsion elaborated with cocoa bean shell flour and walnut oil as fat replacer; GECW100: sample with 100% gelled emulsion elaborated with cocoa bean shell flour and walnut oil as fat replacer. SFA: saturated fatty acids; MUFA: monosaturated fatty acids; PUFA: poluunsaturated fatty acids; AI: atherogenicity index; TI: thrombogenicity index; h/H: hypocholesterolemic/hypercholesterolemic ratios. For each group (raw or cooked), values followed by the same small letter within the same row are not significantly different (*p* > 0.05) according to Tukey’s multiple range test.

**Table 4:** Physic-chemical properties of raw and cooked beef burger elaborated with cocoa bean shell flour and walnut oil emulsion gel as partial and total pork backfat replacers.

	Raw			Cooked		
	CS	GECW50	GECW100	CS	GECW50	GECW100
pH	5.82 ± 0.01 ^a^	5.72 ± 0.02 ^b^	5.64 ± 0.02 ^c^	5.90 ± 0.02 ^a^	5.78 ± 0.04 ^b^	5.68 ± 0.11 ^c^
L*	38.54 ± 4.03 ^b^	42.29 ± 2.07 ^a^	42.43 ± 1.66 ^a^	44.06 ± 3.27 ^a^	39.57 ± 4.06 ^b^	39.01 ± 4.50 ^b^
a*	7.96 ± 2.30 ^a^	7.33 ± 0.88 ^a^	7.56 ± 0.67 ^a^	4.86 ± 0.81 ^a^	5.54 ± 0.66 ^a^	5.47 ± 0.51 ^a^
b*	9.29 ± 1.62 ^b^	11.16 ± 0.90 ^a^	10.86 ± 0.95 ^a^	11.37 ± 1.32 ^a^	9.56 ± 1.51 ^b^	9.24 ± 1.18 ^b^
C*	12.30 ± 2.49 ^b^	13.36 ± 1.13 ^a^	13.25 ± 0.99 ^a^	12.40 ± 1.21 ^a^	11.07 ± 1.45 ^b^	10.77 ± 0.99 ^b^
h*	50.08 ± 6.54 ^b^	56.77 ± 2.43 ^a^	55.12 ± 2.55 ^a^	66.67 ± 4.52 ^a^	59.57 ± 4.44 ^b^	59.05 ± 4.56 ^b^
ΔE	---	4.57 ± 1.72 ^a^	4.36 ± 1.71 ^a^	---	5.50 ± 3.59 ^a^	5.94 ± 4.14 ^a^

CS: control sample elaborated with the traditional formula; GECW50: sample with 50% gelled emulsion elaborated with cocoa bean shell flour and walnut oil as fat replacer; GECW100: sample with 100% gelled emulsion elaborated with cocoa bean shell flour and walnut oil as fat replacer. L*: lightness; a*: redness; b*: yellowness; C*: chroma; h*: hue; ΔE: Total colour differences. For each group (raw or cooked) values followed by the same small letter within the same row are not significantly different (*p* > 0.05) according to Tukey’s multiple range test.

**Table 5:** Cooking characteristics of beef burger elaborated with cocoa bean shell flour and walnut oil emulsion gel as partial and total pork backfat replacers.

	CS	GECW50	GECW100
Cooking loss (%)	28.04 ± 0.79 ^a^	25.44 ± 0.25 ^b^	22.62 ± 1.41 ^c^
Shrinkage (%)	19.77 ± 0.91 ^a^	17.99 ± 1.16 ^b^	17.27 ± 1.03 ^b^
Thickness increase (%)	12.48 ± 0.81 ^a^	10.44 ± 0.33 ^b^	7.14 ± 0.12 ^c^

CS: control sample elaborated with the traditional formula; GECW50: sample with 50% gelled emulsion elaborated with cocoa bean shell flour and walnut oil as fat replacer; GECW100: sample with 100% gelled emulsion elaborated with cocoa bean shell flour and walnut oil as fat replacer. Values followed by the same letter within the same row are not significantly different (*p* > 0.05) according to Tukey’s multiple range test.

## Data Availability

The data presented in this study are available upon request from the corresponding author.
